# Anxiety disorders and adhesive capsulitis: a bidirectional Mendelian randomization study

**DOI:** 10.3389/fimmu.2023.1297477

**Published:** 2024-01-08

**Authors:** Yi Ouyang, Miaomiao Dai

**Affiliations:** ^1^ Department of Joint Surgery, Shunde Hospital, Southern Medical University (The First People’s Hospital of Shunde, Foshan), Foshan, Guangdong, China; ^2^ Department of Ophthalmology, Shunde Hospital, Southern Medical University (The First People’s Hospital of Shunde, Foshan), Foshan, Guangdong, China

**Keywords:** anxiety disorders, adhesive capsulitis, frozen shoulder, Mendelian randomization, causality

## Abstract

**Background:**

Previous epidemiological investigations and related research efforts consistently have outlined an observable association between anxiety disorders and adhesive capsulitis (AC). However, the intricate nature of the causal connection between these entities has yet to be fully clarified. Therefore, this investigative study aims to thoroughly examine and delineate the causal interrelationship between anxiety disorders and AC using a bidirectional, two-sample Mendelian randomization (MR) approach.

**Methods:**

To pursue this inquiry, datasets related to anxiety disorders and AC were meticulously obtained from a publicly accessible genomewide association study. Instrumental variables, in the form of single nucleotide polymorphisms, were subsequently identified, undergoing a rigorous screening process that included intensity adjustment and the amelioration of linkage disequilibrium. The primary analytical tool for scrutinizing causal ramifications was the inverse variance weighting (IVW) methodology, complemented by supplementary analytical techniques such as weighted median, MR-Egger, simple mode, and weighted mode. Additionally, evaluations of heterogeneity and pleiotropy were meticulously conducted. Heterogeneity was assessed using Cochran’s Q-test in conjunction with the IVW and MR-Egger methods, while pleiotropy was appraised through the MR-Egger intercept and MR-PRESSO analysis methods. A leave-one-out analysis was undertaken to enhance the reliability of our findings. Finally, AC was utilized to infer reverse causality concerning the risk of anxiety disorders.

**Results:**

The random effects IVW analysis results yielded statistical significance (*P* = 9.362 × 10^-6^), demonstrating a causal link between anxiety disorders and elevated susceptibility to AC, reflected in an odds ratio of 1.267 (95% confidence interval: 1.141–1.407). Conversely, the inverse MR analysis predominantly produced null findings. Furthermore, sensitivity analyses underscored the robustness of our conclusions.

**Conclusion:**

In summary, our meticulously conducted study unequivocally supports the presence of a causal connection between anxiety disorders and an increased propensity for AC. Unfortunately, the reverse MR analysis failed to provide compelling evidence indicative of a reciprocal genetic causative relationship between AC and anxiety disorders.

## 1 Introduction

Adhesive capsulitis (AC), also known as frozen shoulder, is a pathology characterized by progressive, spontaneously occurring shoulder pain associated with a loss of passive and active joint movement (1). The prevalence of AC in the general population ranges between 2% and 5% (2). It is more common in females and individuals aged between 40 and 60 years (3). The literature on recovery from AC remains controversial. Some studies describe it as a self-limiting pathology, while others suggest the need for care, with symptoms resolving over a period ranging from a few months to two years (4–7). Additionally, while some patients report complete symptom resolution, others experience a residual range of motion impairments and pain (8). Despite the proposed three-phased evolution of the disease (freezing, frozen, and thawing), there is insufficient strong evidence supporting this subclassification in terms of prognostic or diagnostic value (9). Diabetes mellitus is identified as a major risk factor associated with AC, yet the exact etiology of AC remains unknown (10).

In recent decades, studies have indicated that psychological disorders may be probable risk factors in explaining the etiology of musculoskeletal diseases (11, 12). Regarding the association between psychological parameters and AC, initial research focused on personality disorders, with the concept of a “frozen shoulder in a frozen personality” gaining recognition (13–15). AC is linked to physical disability and reduced quality of life (16, 17). Furthermore, AC negatively impacts work productivity, with working-age adults experiencing AC more likely to be on long-term sick leave compared to those without the condition (18). In this context, AC may be a risk factor for anxiety disorders and poor mental health, as several studies have reported relatively frequent anxiety symptoms in individuals with AC (3, 16, 17). However, most current research on the relationship between anxiety disorders and AC relies on observational studies, susceptible to the interference of confounding factors. Therefore, the causal relationship between anxiety disorders and AC remains unclear, necessitating additional research to elucidate the intricate and enigmatic connection between the two.

Mendelian randomization (MR) is an alternative statistical approach used to assess causality when randomized controlled trials (RCTs) are not feasible (19). By utilizing genetic variants as instrumental variables (IVs) derived from large-scale genome-wide association studies (GWAS), MR can offer insights into causal factors for complex diseases (20). The random assortment of genetic variants at meiosis makes the MR design a natural analog of an RCT, thereby reducing the likelihood of bias compared to observational research (21). Furthermore, reverse causality is less likely due to the one-directional information pathway from DNA sequence to phenotypes (genotype formation prior to disease onset). Therefore, in this study, we aimed to conduct a two-sample bidirectional MR study to dissect the potential causal association of anxiety disorders with AC, leveraging GWAS data.

## 2 Methods

### 2.1 Study design

**Figure 1:**
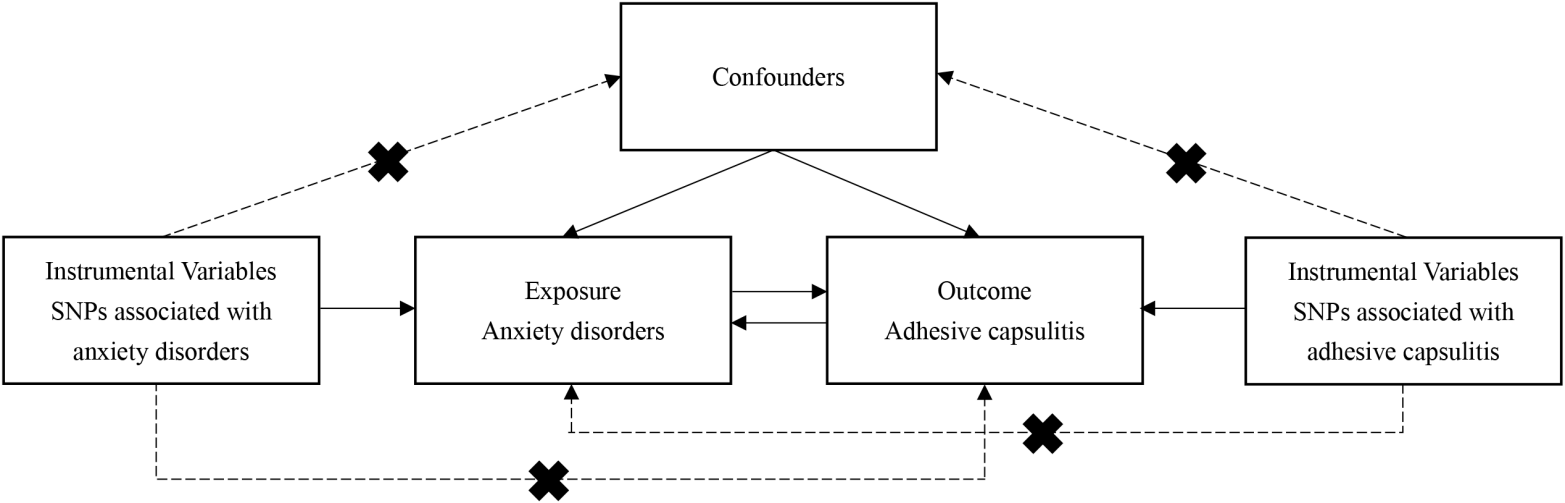
Schematic representation of analytical workflow and core hypothesis in the two-sample Mendelian randomization (MR) study. Three fundamental assumptions underpin the MR analysis: Assumption 1 — The instrumental variable, representing genetic variation, must be strongly associated with the exposure (anxiety disorders or AC). Assumption 2 — Confounders between the exposure and outcome should not influence the genetic variation. Assumption 3 — Genetic variation should exclusively impact the outcome (anxiety disorders or AC) through the exposure, independently of alternative pathways. MR, Mendelian randomization; AC, adhesive capsulitis.

To explore the underlying causal dynamics between anxiety disorders and AC, a bidirectional two-sample MR inquiry was conducted. The MR analysis relies on three fundamental assumptions: Assumption 1 — The IV, representing genetic variation, must be strongly associated with the exposure (anxiety disorders or AC). Assumption 2 — Confounders between the exposure and outcome should not influence the genetic variation. Assumption 3 — Genetic variation should exclusively impact the outcome (anxiety disorders or AC) through the exposure, independently of alternative pathways (refer to **Figure 1**). Due to the use of openly accessible repositories, ethical sanction or participant informed consent was not required.

### 2.2 Data sources

Summary data from GWAS relevant to anxiety disorders were obtained from the FinnGen Consortium’s designated repository (https://r9.finngen.fi/) (22). The analysis focused on the “anxiety disorders (more control exclusions)” phenotype subset within the database, comprising a cohort of 40,191 cases and 277,526 controls. Anxiety disorders, encompassing a spectrum of psychiatric maladies characterized by anxious affect and fear, often coupled with somatic manifestations, were the primary focus. Similarly, GWAS summary data for AC were acquired from the FinnGen Consortium, including 5,538 cases and 4,901 controls.

### 2.3 IV selection

To identify single nucleotide polymorphisms (SNPs) associated with the exposure, a more permissive statistical threshold (*P* < 5 × 10^−6^) was adopted, allowing for the inclusion of a broader array of IVs correlated with the exposure of interest (23). An algorithmic clumping approach, with a linkage disequilibrium threshold of *r^2^* > 0.1 within a genomic interval spanning 5,000 kilobases, was employed to ensure the statistical independence of integrated SNPs as IVs (24, 25). Supplementary validation involved assessing the viability of each genetic instrument through the computation of the *F*-statistic, represented as *β* ^2^/*se* ^2^. An *F*-statistic exceeding 10 was set as a prerequisite for downstream investigations to mitigate weak IV bias (26). To minimize the influence of pleiotropy, SNPs implicated in confounding processes and outcome determinants were excluded using the Phenoscanner V2 resource. The pivotal role of diabetes and rotator cuff injury as confounders in AC investigation and depression as a confounder in anxiety disorder exploration was acknowledged. This procedural framework ensured that the IVs uniquely impacted the exposure, avoiding alternative causal pathways. Subsequently, the exposure and outcome datasets were harmonized, with the exclusion of SNPs featuring palindromic attributes and intermediate allele frequencies to ensure congruence of effect alleles.

### 2.4 Statistical analysis

A combination of statistical methodologies, including inverse variance weighting (IVW), MR-Egger regression, weighted median, simple mode, and weighted mode, was employed to elucidate the genetic underpinnings linking the exposure and outcome phenomena. The IVW method, a traditional approach, amalgamated Wald ratio estimations of causal impact derived from diverse SNPs using a meta-analytic paradigm. Implicit in the IVW framework was the assumption of the validity of all constituent SNPs as instrumental agents, yielding estimations of maximum fidelity and classifying IVW as the principal analytical modality (27). Visual representation of outcomes was provided through scatter plots and funnel plots. Heterogeneity in associations was assessed using Cochrane’s *Q* test, while funnel plot symmetry illuminated heterogeneity. The plausibility of pleiotropy was evaluated through the MR-Egger intercept test and the MR pleiotropy residual sum and outliers (MR-PRESSO) global test, the latter not only detecting outliers but also providing recalibrated estimations post outlier exclusion. The sensitivity of findings was tested through a leave-one-out approach, exploring the influence of individual influential SNPs and yielding insights into the collective impact of the remaining genetic instruments. Statistical analyses were conducted using R v4.3.1 and the TwoSampleMR package, with statistical significance set at *P* < 0.05.

## 3 Results

### 3.1 Causal effects of anxiety disorder on AC

#### 3.1.1 IV selection

In the initial assembly of 131 SNPs, a meticulous screening process based on significant linkage (*P* < 5 × 10^-6^, *F*-value > 10) and necessary independence (*r* ^2^ < 0.1) within a 5,000-kilobase physical span was conducted. **Supplementary Table 1** provides a detailed exposition of *F*-values. Employing Phenoscanner V2, SNPs associated with outcomes and confounders were selectively removed, leading to the exclusion of *rs200465*, *rs17205528*, *rs4702*, and *rs28412876*. The dataset was then further refined by excluding 21 palindromic SNPs with intermediate allele frequencies. The MR-PRESSO procedure identified and removed one outlier SNP, resulting in a validated set of 105 SNPs designated as instrumental agents for subsequent MR analysis.

#### 3.1.2 MR analysis

**Figure 2:**
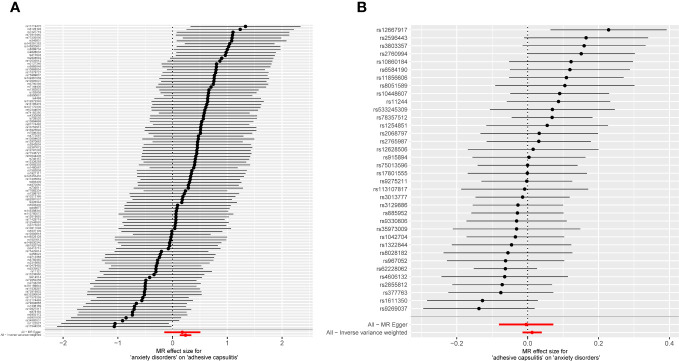
Forest plot displaying MR analyses for individual variants, incorporating MR-Egger and inverse-variance weighting approaches. **(A)** Exposure: anxiety disorders; outcome: AC. **(B)** Exposure: AC; outcome: anxiety disorders. MR, Mendelian randomization; AC, adhesive capsulitis.

**Figure 3:**
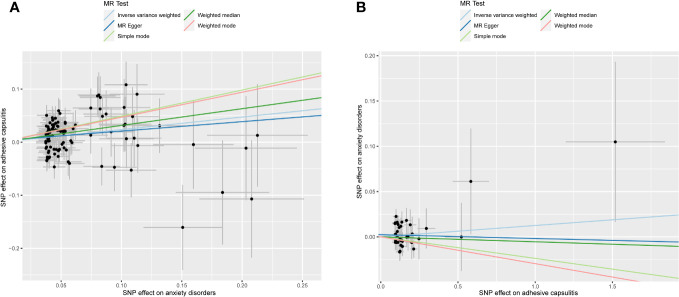
Scatter plot illustrating MR analyses. The slopes of the lines represent causal estimates derived from five distinct methodologies, including inverse-variance weighting, MR-Egger regression, weighted median, simple mode, and weighted mode. **(A)** Exposure: anxiety disorders; outcome: AC. **(B)** Exposure: AC; outcome: anxiety disorders. MR, Mendelian randomization; AC, adhesive capsulitis.

An exploration of the genetic relationship between anxiety disorders and AC utilized the random-effects IVW methodology. The findings revealed anxiety disorders as a clear risk factor for AC (*P* = 9.362 × 10^-6^, odds ratio [OR] [95% confidence interval (CI)] = 1.267 [1.141–1.407]), as illustrated in **Figure 2A**. This genetic causative association was further supported by the consistent validity of the weighted median, simple mode, and weighted mode techniques. The comprehensive results of these analytical modalities are visually presented in **Figure 3A** and summarized in **Table 1**.

**Table 1:** Association between anxiety disorders and AC by MR models.

Method	*n*SNP	OR	95% CI	*P*-value
Inverse variance weighted	105	1.267	1.141–1.407	9.362 × 10^-6^
MR Egger	105	1.195	0.860–1.661	0.292
Weighted median	105	1.371	1.193–1.575	8.314 × 10^-6^
Simple mode	105	1.635	1.076–2.483	0.023
Weighted mode	105	1.601	1.066–2.405	0.025

CI, confidence interval; SNP, single nucleotide polymorphism; MR, Mendelian randomization; OR, odds ratio; AC, adhesive capsulitis.

#### 3.1.3 Sensitivity analysis and visual depiction

**Figure 4:**
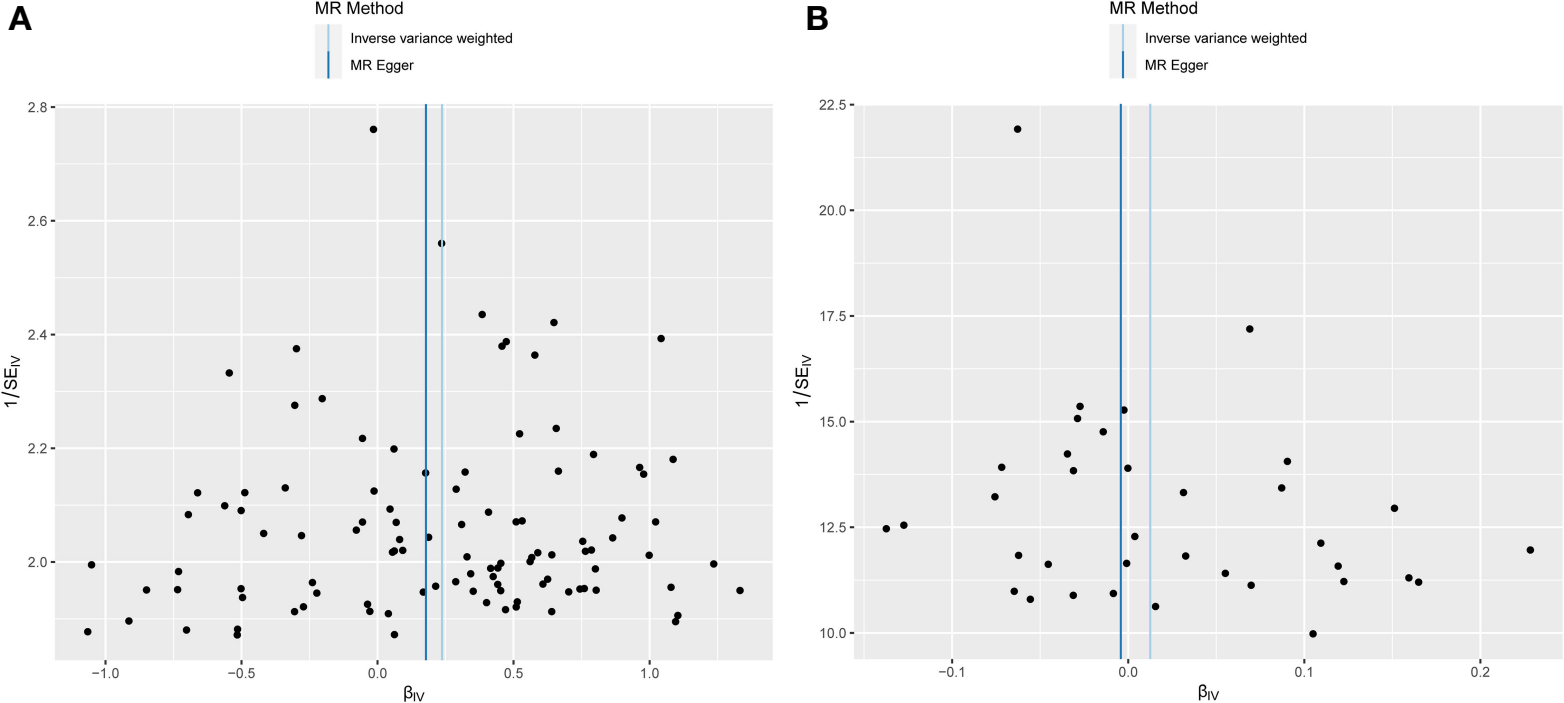
Funnel plot depicting MR analysis results. The absence of evident asymmetry within the plot indicates homogeneity in the association. **(A)** Exposure: anxiety disorders; outcome: AC. **(B)** Exposure: AC; outcome: anxiety disorders. MR, Mendelian randomization; AC, adhesive capsulitis.

**Figure 5:**
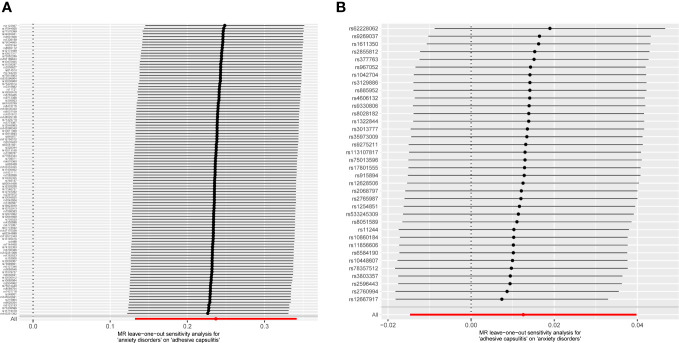
MR leave-one-out analyses. No instances were observed where the exclusion of a specific single nucleotide polymorphism induced a significant alteration in the outcome, affirming the stability of this association. **(A)** Exposure: anxiety disorders; outcome: AC. **(B)** Exposure: AC; outcome: anxiety disorders. MR, Mendelian randomization; AC, adhesive capsulitis.

Examination of MR-Egger regression (Cochran’s *Q* = 133.044, *P* = 0.025) and IVW analysis (Cochran’s *Q* = 133.220, *P* = 0.028) revealed no indications of heterogeneity. Simultaneously, the symmetry of SNPs was distinctly evident in the funnel plot (**Figure 4A**). The robustness of this association remained unblemished by horizontal pleiotropy, as affirmed by the MR-intercept test (*P* = 0.713). Furthermore, the leave-one-out analysis presented a consistent narrative, untouched by any single SNP exerting disproportionate influence over the trajectory of MR outcomes (**Figure 5A**), thereby lending strength and consistency to the findings.

### 3.2 Reverse MR analysis

#### 3.2.1 IV selection

The preliminary cohort of 47 SNPs constituted the initial selection of IVs. These genetic markers emerged successfully from a rigorous winnowing process, where SNPs underwent thorough evaluation for significant linkage (*P* < 5 × 10^-6^, *F*-value > 10) and necessary independence (*r^2^* < 0.1) within a 5,000-kilobase span, resulting in their inclusion. **Supplementary Table 2** provides a comprehensive repository of *F*-values. Adhering strictly to predefined criteria, SNPs associated with both outcomes and potential confounders were excluded from the analytical sphere through the discerning capability of Phenoscanner V2, with two confounding SNPs proving resilient. While the alignment of exposure and outcome data was commendable, it led to the exclusion of nine palindromic SNPs with intermediate allele frequencies. Ultimately, a set of 36 SNPs was designated as IVs, poised for future exploration through MR analysis.

#### 3.2.2 MR analysis

In unraveling the genetic intertwining between AC and anxiety disorders, the eminent random-effects IVW modality took precedence in the analytical realm. Within the canvas of IVW analysis, no discernible causal impact of AC on susceptibility to anxiety disorder was evident (*P* = 0.366, OR [95% CI] = 1.013 [0.985–1.041], **Figure 2B**). Simultaneously, evidence from MR-Egger, weighted median, simple mode, and weighted mode techniques harmoniously concurred in affirming the conclusions. An illustrative panorama of our MR investigation, showcasing the accomplishments of the five methodologies, is presented in **Figure 3B** and summarized in **Table 2**.

**Table 2:** Association between AC and anxiety disorders by MR models.

Method	*n*SNP	OR	95% CI	*P*-value
Inverse variance weighted	36	1.013	0.985–1.041	0.366
MR Egger	36	0.996	0.922–1.075	0.915
Weighted median	36	0.995	0.961–1.030	0.760
Simple mode	36	0.977	0.903–1.057	0.558
Weighted mode	36	0.971	0.906–1.040	0.406

CI, confidence interval; SNP, single nucleotide polymorphism; MR, Mendelian randomization; OR, odds ratio; AC, adhesive capsulitis.

#### 3.2.3 Sensitivity analysis and visual exposition

The MR-Egger regression milieu (Cochran’s *Q* = 41.168, *P* = 0.186) and the stronghold of IVW analysis (Cochran’s *Q* = 41.422, *P* = 0.211) revealed a conspicuous absence of heterogeneity. Simultaneously, the harmonious symphony depicted by the funnel plot mirrored the symmetrical disposition of SNPs (**Figure 4B**). The effectiveness in countering horizontal pleiotropy was resoundingly affirmed through the MR-intercept test (*P* = 0.650). A paragon of meticulousness, the leave-one-out inquiry, lucidly demonstrated the imperviousness of individual SNPs to usher consequential perturbations within the realm of MR outcomes (**Figure 5B**), thereby bestowing an unwavering mantle of reliability and consistency upon our revelations.

## 4 Discussion

Employing a bidirectional two-sample MR approach with publicly available GWAS summary data, we aimed to determine the presence of a bidirectional causal relationship between anxiety and AC. Our two-sample MR analysis revealed an increased likelihood of AC development as a consequence of anxiety. However, our findings did not provide evidence supporting a causal link between genetic susceptibility to AC and an elevated risk of anxiety.

The existing literature has highlighted a growing association between anxiety and AC. A comprehensive systematic review indicated that patients with AC reporting comorbid anxiety and depression exhibited heightened pain perception, reduced functionality, and a diminished quality of life compared to their healthy counterparts. Nevertheless, it remains unclear whether these psychological facets were preexisting or developed following an AC diagnosis (28). In a cross-sectional study, Fernandes et al. identified a “doubtful” positive association between anxiety symptoms and AC (29). Bagheri et al., in another cross-sectional investigation, assessed pain, disability, quality of life, and associated factors in patients with AC. Their findings suggested that patients with AC exhibited elevated rates of pain and disability alongside diminished quality of life compared to the general population. Furthermore, they observed a stronger correlation between pain, disability, and the mental component of quality of life with psychological factors such as anxiety, as opposed to physical or personal parameters such as age, gender, education, or range of motion (16). Ebrahimzadeh et al., in a cross-sectional study involving 120 patients with idiopathic AC, reported that 26.7% of the cohort experienced anxiety, with those afflicted by anxiety enduring more pronounced pain and disability in the affected limb (10). In a cross-sectional investigation by Ding et al., the risk of anxiety in primary patients with AC was evaluated, and the relationship between psychological disorders and disease status was explored. Their study indicated that 24.2% of AC participants were at high risk for anxiety, and patients with AC and anxiety manifested increased shoulder pain, greater restrictions in range of motion, and a higher prevalence of sleep disturbances (3). Mello et al., through a retrospective analysis of 1,983 patients with shoulder disorders during the COVID-19 pandemic, identified a 2.41-fold surge in AC cases compared to the preceding year. Notably, patients with anxiety exhibited a substantially heightened 14-fold risk of developing AC during the pandemic (30). In a prospective study, Toprak et al. assessed 76 patients with AC and 72 healthy controls, examining anxiety, depression, sleep quality, and quality of life in patients with AC relative to controls. Their findings indicated a substantial prevalence of anxiety in patients with AC, suggesting potential benefits in incorporating psychiatric evaluation into AC treatment regimens (17).

However, it is crucial to acknowledge that the aforementioned studies were predominantly cross-sectional, lacking a clear chronological order, which makes establishing causality challenging. Additionally, these prior observational inquiries faced limitations such as small sample sizes, difficulties in avoiding reverse causation, and the impact of confounding variables. In contrast, our investigation employed a more robust study design, allowing for a more effective elucidation of the causal relationship between exposure and outcome through the use of a bidirectional two-sample MR analytical approach. Aim et al. prospectively enrolled 77 consecutive patients undergoing arthroscopic rotator cuff repair (ARCR) and revealed that preoperative anxiety constitutes a risk factor for AC development following ARCR, aligning with our study findings (31).

Our bidirectional two-sample MR study offers several advantages. First, we utilized the MR analytical framework, employing SNPs with substantial association strength (*F*-statistic > 10) as IVs, simulating an experimental design similar to RCTs. RCTs, while traditionally providing high evidentiary support in clinical practice, are hindered by limitations such as high costs and limited sample sizes (32). In contrast, our MR approach adeptly addresses pitfalls associated with reverse causation and confounding variables. Second, all data used in our analysis originated from the GWAS database, exclusively comprising European population samples, minimizing confounding effects due to population heterogeneity. Third, the outcomes of our investigation have potential implications for healthcare policy, as understanding a causal link between anxiety and AC may influence public health policies related to prevention and therapeutic interventions.

However, our study has several notable limitations. First, our MR analysis exclusively included individuals of European ancestry, necessitating further exploration to determine the generalizability of our findings to diverse ancestral populations. Second, due to the limited availability of relevant GWAS summary data, our investigation did not explore the influence of gender, age, and body mass index stratification on psychiatric disorders. Lastly, to enhance the number of SNPs used in our MR analyses, we implemented a significance threshold for the *P*-value at 5 × 10^-6^, resulting in certain IVs with relatively modest proportions of explained variance.

## 5 Conclusion

Our findings offer empirical support for a causal association between anxiety disorders and AC, potentially carrying significant implications for clinical decision-making regarding the management of anxiety disorders in patients diagnosed with AC. It is crucial to note that our results did not provide evidence for a causal influence of AC on anxiety disorders. Consequently, additional investigations are warranted to elucidate the precise impact of AC on anxiety disorders.

## Data availability statement

The original contributions presented in the study are included in the article/**Supplementary Material**. Further inquiries can be directed to the corresponding author.

## Author contributions

YO: Conceptualization, Data curation, Formal analysis, Writing – original draft. MD: Supervision, Validation, Visualization, Writing – review & editing.
